# Hidradenitis Suppurativa in the United States: Insights From the National Inpatient Sample (2008-2017) on Contemporary Trends in Demographics, Hospitalization Rates, Chronic Comorbid Conditions, and Mortality

**DOI:** 10.7759/cureus.24755

**Published:** 2022-05-05

**Authors:** Amie Patel, Anjali Patel, Dhanshree Solanki, Uvesh Mansuri, Aanandita Singh, Purnima Sharma, Shantanu Solanki

**Affiliations:** 1 Medicine, New York Institute of Technology College of Osteopathic Medicine, Old Westbury, USA; 2 Medicine, University of Texas at Austin, Austin, USA; 3 Hospital Administration, Rutgers University, New Brunswick, USA; 4 Medicine, MedStar Good Samaritan Hospital, Baltimore, USA; 5 Medicine, Sri Guru Ram Das Institute of Medical Sciences & Research, Amritsar, IND; 6 Medicine, Texas Tech University Health Sciences Center, El Paso, USA; 7 Hospital-Based Medicine, Independent Researcher, Hoboken, USA

**Keywords:** hs hospitalizations, mortality, comorbidities, length of stay, hidradenitis suppurativa

## Abstract

Background

Hidradenitis suppurativa (HS) is a clinical condition characterized by the formation of painful lumps under the skin. It often affects intertriginous areas like armpits and groin. There is a paucity of contemporary data on patient and hospital-level characteristics of HS in the United States.

Methods

We analyzed the Nationwide Inpatient Sample (NIS) for retrospective analysis to calculate the frequency and yearly rates of HS hospitalizations, demographic variations, rates of comorbidities, and length of stay.

Results

The rate of hospitalizations with HS as a primary diagnosis increased from 7.9 per 100,000 all-cause hospitalizations in 2008 to 11.6 per 100,000 all-cause hospitalizations in 2017 (p < 0.0001). The mean age ± standard error of hospitalized patients was 39.5 ± 0.2 years. The age group of 18-34 years was the most affected. Women showed a higher preponderance of the disease than men (56.6% vs. 43.5%, p < 0.0001). The Black race was the most affected out of all the racial groups (59.9%). Most hospitalizations were in large, urban teaching hospitals. Hypertension (34.9%), skin and subcutaneous tissue infections (26.5%), and diabetes mellitus (25.9%) were the most common comorbidities. Out of the total hospitalizations with HS, 12.7% were found to have a major or extreme loss of function and 3.5% were at a major or extreme likelihood of dying.

Conclusions

HS disproportionately affects young adults, women, and Black patients. A significant proportion of these patients are at a major risk of major loss of bodily function or death. Prospective studies are needed to identify the risk factors for hospitalizations in these patient populations and devise appropriate prevention and treatment strategies.

## Introduction

Hidradenitis suppurativa (HS) is a chronic inflammatory skin condition that primarily affects axillary, inframammary, inguinal, perianal, and perineal regions [1]. The pathogenesis of HS is not well understood. Follicular occlusion followed by bacterial infection is believed to be the most likely inciting event [2]. Various genetic, hormonal, and environmental factors have been implicated in the development of HS [3,4]. These include smoking, obesity, high serum levels of certain interleukins, presence of gamma-secretase mutations, and alterations in progesterone and estrogen levels [5-7]. Its clinical course is unpredictable and can vary from nodules, papules, pustules, abscesses, sinus tracts, and scars [8,9]. HS flares are common and can occur weekly in more than half of the patients affected with it [10]. It significantly impacts their psychosocial and sexual health while diminishing their quality of life [11,12]. The prevalence of HS is around 0.1% in the US population [13]. Anzaldi et al. reported that the inpatient hospitalization rate for HS increased by 60% from 1999 to 2014 [14]. However, the inpatient data on patient and hospital-level characteristics, the severity of illness, and rates of common comorbidities associated with this chronic disease remain underreported.

## Materials and methods

Data source

The Nationwide Inpatient Sample (NIS) is the largest publicly available inpatient database in the US. It was developed for the Healthcare Cost and Utilization Project (HCUP) and contains data on more than seven million hospital stays [15]. It is a de-identified database, which has safeguards in place to protect the privacy of patient data. It can be utilized to produce US regional and national estimates of diseases and their outcomes [15,16].

Description of diagnoses, codes, and variables

The first listed diagnosis is the principal diagnosis. It is the disease responsible for a patient’s admission to the hospital for care [17]. The diseases co-existing at the time of hospital admission or those that develop during the patient’s hospital stay are referred to as secondary diagnoses. HCUP records report diagnoses based on the International Classification of Diseases, Ninth and Tenth Revision, Clinical Modification (ICD-9-CM and ICD-10-CM). It is a standardized coding system, which defines diseases in a hierarchical system using alphanumeric codes [18]. The Clinical Classifications Software (CCS) helps to cluster clinical diagnoses into different categories [19]. The relevant ICD-9 and ICD-10 codes can be found in Supplement 1. The description of variables, i.e., region, location of the hospital, median household income, insurance status, bed size, and severity of illness, can be found in Supplement 2. All Patient Refined Diagnosis Related Group (APR-DRG) is a severity measurement system developed by 3M Health Information Systems [20]. We used APR-DRG to determine the severity of illness and risk of mortality. Under the Health Insurance Portability and Accountability Act (HIPAA), institutional review board (IRB) approval is not required when this dataset is used for analysis [21].

Statistical analysis

SAS 9.3 software (SAS Institute, Cary, NC) was used for the entirety of our analysis. HCUP provides weighted values to produce nationally representative estimates [22]. We used the χ2 test to compare the categorical variables. This statistical approach is well documented, and it has been used in many peer-reviewed NIS-based studies [23-25].

## Results

Trends in HS hospitalizations

**Figure 1:**
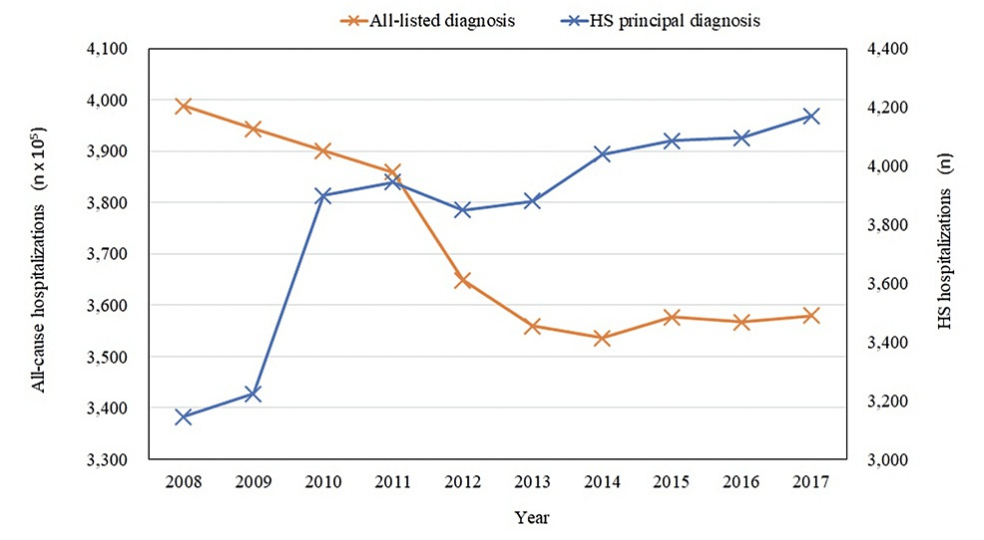
Yearly rate of hospitalizations HS: hidradenitis suppurativa.

A total of 38,331 hospitalizations with a primary diagnosis of HS were extracted from the database. There were 3,145 hospitalizations in 2008 and 4,170 hospitalizations in 2017. The rate of HS hospitalizations showed an upward trend during the study period whereas the all-cause hospitalizations showed a downward trend (Figure 1). The rate of HS hospitalizations increased from 7.9 per 100,000 all-cause hospitalizations in 2008 to 11.6 per 100,000 all-cause hospitalizations in 2017 (p < 0.0001) (Table 1).

**Table 1:** Trends in HS and all-cause hospitalizations HS: hidradenitis suppurativa.

	Hospitalizations with HS as the principal diagnosis	All-cause hospitalizations	HS per 100,000 hospitalizations
2008	3,145	39,885,120	7.9
2009	3,223	39,434,956	8.2
2010	3,898	39,008,298	10.0
2011	3,945	38,590,733	10.2
2012	3,850	36,484,846	10.6
2013	3,880	35,597,792	10.9
2014	4,040	35,358,818	11.4
2015	4,085	35,769,942	11.4
2016	4,095	35,675,421	11.5
2017	4,170	35,798,453	11.6
Total	38,331	371,604,379	10.3

Patient and hospital-level characteristics

**Figure 2:**
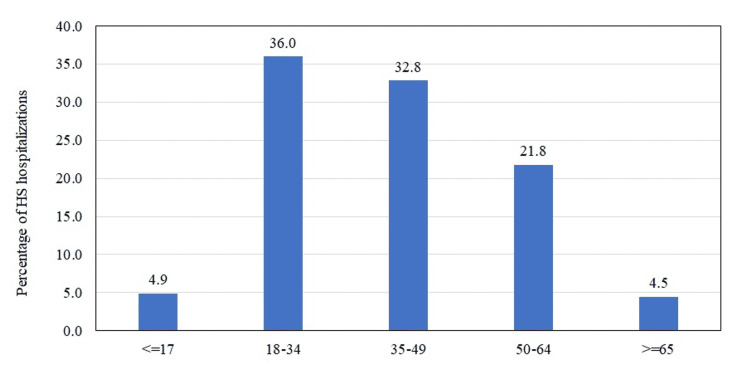
Age-wise distribution of HS hospitalizations HS: hidradenitis suppurativa.

**Figure 3:**
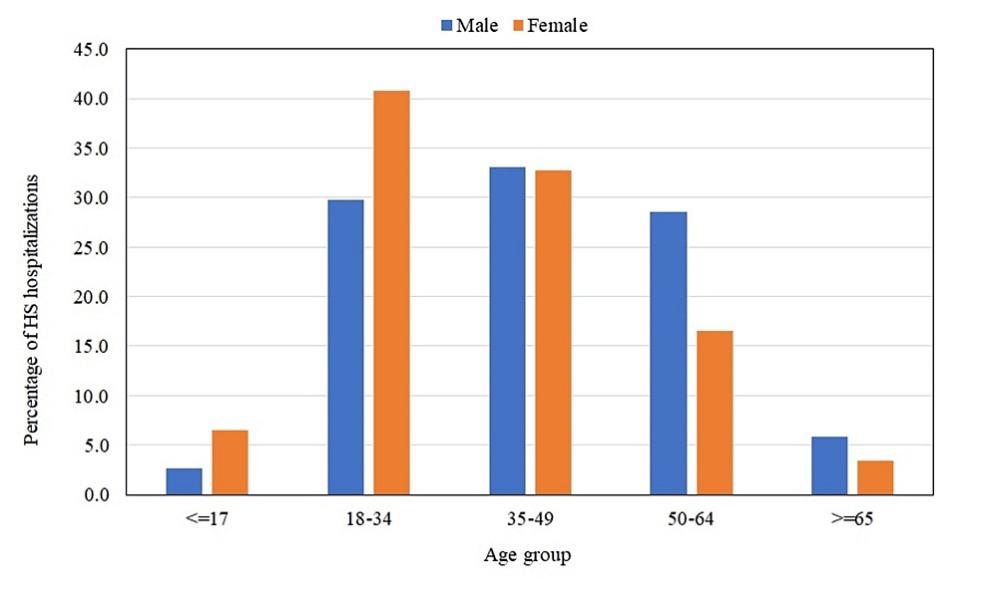
Male-to-female (M:F) ratio for HS hospitalizations HS: hidradenitis suppurativa.

The mean age ± standard error (SE) of hospitalized patients was 39.5 ± 0.2 years. The age group of 18-34 years was the most affected (36%) (Figure 2, Table 2). Patients above 65 years of age were least affected by HS (4.5%) (Figure 2, Table 2). Women showed a higher preponderance of the disease than men (56.6% vs. 43.5%, p < 0.0001) (Table 2). The overall male-to-female (M:F) ratio during the study period was 1:1.30 (Table 3). M:F ratio was highest in patients less than 17 years of age (1:2.42) followed by the age group of 18-34 years (1:1.37). The ratio was reversed for patients aged 35 years and above, leading to a male preponderance for HS (Figure 3). The Black race was the most affected (59.9%) followed by Whites (28.5%). Asians and Native Americans were the least affected. Patients with the lowest median household income quartile had the highest preponderance of HS and those belonging to the highest quartile had the lowest percentage of hospitalizations (44.9% vs. 11.7%, p < 0.0001). Most patients were insured by Medicare/Medicaid (54.8%). The mean length of stay during the study period was 5.8 ± 0.1 (days ± SE).

**Table 2:** Baseline characteristics of HS hospitalizations HS: hidradenitis suppurativa; HMO: health maintenance organization; LOS: length of stay; SE: standard error.

Variable		P-value
Number of observations (n)	38,331	
Mean age (years ± SE)	39.5 ± 0.2	
Age in years (%)		<0.0001
<=17	4.9	
18-34	36.0	
35-49	32.8	
50-64	21.8	
>=65	4.5	
Gender (%)		<0.0001
Male	43.5	
Female	56.6	
Race (%)		<0.0001
White	28.5	
Black	59.9	
Hispanic	7.7	
Others	4.0	
Region (%)		<0.0001
Northeast	19.1	
Midwest	22.7	
South	47.1	
West	11.1	
Location (%)		<0.0001
Rural	6.6	
Urban nonteaching	20.7	
Urban teaching	72.7	
Median household income (%)		<0.0001
Quartile 1	44.9	
Quartile 2	24.4	
Quartile 3	19.1	
Quartile 4	11.7	
Payment (%)		<0.0001
Medicare/Medicaid	54.8	
Private including HMO	30.8	
Uninsured/self-pay	14.4	
Bed size		<0.0001
Small	13.1	
Medium	24.4	
Large	62.5	
Mean LOS (days ± SE)	5.8 ± 0.1	

**Table 3:** Male-to-female (M:F) distribution of HS hospitalizations HS: hidradenitis suppurativa.

	M:F ratio
Overall	1:1.30
<=17	1:2.42
18-34	1:1.37
35-49	1:0.99
50-64	1:0.58
>=65	1:0.58

Hospital-level characteristics

These characteristics are depicted in Table 3. Southern states had the highest percentage of HS hospitalizations whereas the western US had the lowest percentage of hospitalizations (47.1% vs. 11.1%, p < 0.0001). Most HS hospitalizations were identified in urban teaching hospitals and the least were in rural hospitals (72.7% vs. 6.6%, p < 0.0001). Large hospitals showed the highest percentage of HS hospitalizations (62.5%).

Comorbidities and severity of illness

Hypertension (34.9%), skin and subcutaneous tissue infections (26.5%), and diabetes mellitus (25.9%) were the most common comorbidities (Table 4). Other common co-existing conditions were morbid obesity, connective tissue disorder (7.9%), and cancer (4.8%). Out of the total HS hospitalizations, 12.7% were found to have a major or extreme loss of function and 3.5% were at a major or extreme likelihood of dying (Table 4).

**Table 4:** Comorbidities and severity of illness associated with HS hospitalizations HS: hidradenitis suppurativa; APR-DRG: All Patient Refined Diagnosis Related Groups.

Comorbidities	Percentage	n
Cancer	4.8	1,823
Connective tissue disorder	7.9	3,026
Chronic obstructive pulmonary disease	4.2	1,622
Congestive heart failure	3.1	1,203
Coronary artery disease	4.8	1,855
Diabetes mellitus	25.9	9,925
Hypertension	34.9	13,389
Morbid obesity	12.3	4,731
Skin and subcutaneous tissue infections	26.5	10,171
Severity of illness	Percentage	n
Percentage hospitalizations with APR-DRG severity illness score: major or extreme loss of function	12.7	4,856
Percentage hospitalizations with APR-DRG risk of mortality score: major or extreme likelihood of dying	3.5	1,356
Mortality rate	0.04	14

## Discussion

This was a retrospective study of contemporary trends of HS using data from the NIS. This analysis showed a significant increase (46.8%) in HS hospitalizations per all-cause hospitalizations from 2008 to 2017. This upward trend follows the pattern of previous increasing rates of HS, such as those found in a Portuguese retrospective database study, as well as a cross-sectional study performed using the NIS database (from 1999 to 2014) [14,26]. HS is diagnostically challenging and is commonly misdiagnosed [27]. However, there has been an increase in HS research within the past two decades, an increase in published papers related to HS, and emerging treatments as well [28]. With greater attention to HS and increasing therapies, there may be a correlation between diagnosis and hospitalization rate; however, further studies must be conducted to analyze this association.

In our study, HS was found to be most prevalent in individuals aged 18-34 years, with the mean age ± SE of hospitalization being 39.5 ± 0.2 years. In a 2017 sex- and age-adjusted population analysis of HS prevalence in the US, HS was also found to be most prevalent amongst patients from ages 30 to 39 years, with similar prevalence from ages 18 to 29 years and 40 to 49 years [13]. HS can take approximately 12 years or longer to diagnose, with the age of onset being after puberty and before 40 years [27]. Given the delayed diagnosis time and age of onset, it is reasonable that the greatest prevalence of HS would be between 18 and 34 years of age. Other factors that affect the age range of prevalence include accessibility and availability of experienced diagnosticians (primary care physicians, dermatologists, emergency department physicians, etc.), patient autonomy in seeking care, and misdiagnosis.

Our study also found a significantly higher predominance of HS in women than in men (p < 0.0001), which could be a result of higher medical utilization by women [29]. Several studies have found that HS is more prevalent in females compared to males [13,14,26,27,30,31]. In a population-based study by Shahi et al., it was found that the highest prevalence of HS was amongst women aged 20-29 years, while for men, the highest prevalence was between 60-69 years [31]. This study had limited generalizability due to the demographic of subjects being limited to a predominantly white county in Southeast Minnesota [31]. However, it is important to note that we believe that the results of our study may be generalized to the US population due to the diversity of cohort, sample size, and standardization of disease estimates. Our results indicate that females had the highest preponderance of HS in the age group of 18-34 years, while male preponderance occurred for patients aged 35 years and above. The cause of HS is likely multifactorial with hormone-mediated factors potentially playing a key role [27,32]. Increased prevalence in females aged 18-34 years may in part be a result of hormone fluctuation due to menstruation, pregnancy, and lactation, challenges unique to women [32]; however, determination of this correlation warrants further study.

Furthermore, our results reveal that those of the Black race were affected most, which is concurrent with studies indicating that HS disproportionately affects African Americans when compared to White patients [13,33,34]. African populations have limited access to effective medical care, including dermatologists who are experienced in diagnosing and treating HS [33]. This may play a factor in increased cases of HS in those of the Black race, along with a higher prevalence of the comorbidities of metabolic syndrome and depression [33]. While the development of HS may have genetic components, no association between genetics and HS can be made due to a lack of research and literature, warranting further studies amongst the various racial subgroups affected by HS [33].

We found that patients with the lowest median household income quartile had the highest prevalence of HS, while those in the highest quartile had the lowest percentage of hospitalizations. In the US, HS has been shown to be more likely in patients of low socioeconomic status (LSES) [35]. HS is a chronic inflammatory skin condition that requires prompt diagnosis and treatment, regular medical care, and lifestyle management [27]. HS patients of LSES face challenges related to the accessibility of healthcare, which complicates disease management and progression [36]. Such challenges include, but are not limited to, attending outpatient appointments; paying for co-payments, medications, wound care supplies, treatments, and therapies; availability of community resources and experienced HS healthcare providers; and transportation to hospitals, clinics, and physicians’ offices, etc. [36]. These challenges exacerbate the high prevalence of HS in populations of LSES, hindering timely diagnosis and treatment of the disease. Our results also indicate that most patients (54.8%) were insured by Medicare/Medicaid, consistent with data gathered in a study by Marvel et al. [37].

Our results demonstrate southern states of the US have the highest percentage of HS hospitalizations, with the western US having the lowest percentage of hospitalizations. These findings are concordant with geographic patterns found in a study characterizing HS inpatient hospitalizations in the US by Anzaldi et al. [14]. Anzaldi et al. found that 29% of HS discharges (2012-2014) were from the South Atlantic census division (Delaware, Maryland, Virginia, West Virginia, North Carolina, South Carolina, Georgia, Florida, and the District of Columbia) [14]. On the other hand, the Mountain division, which forms a large portion of the western US, accounts for only 3% of HS discharges [14]. The southern US, where there is the highest percentage of HS hospitalizations, overlaps geographically with an elevated crude prevalence of racial distribution of African Americans and an elevated crude prevalence of obesity, both risk factors associated with HS [14].

Analysis of the NIS data also revealed that a significant majority (72.7%) of HS hospitalizations were identified in urban teaching hospitals while the least hospitalizations (6.6%) were in rural hospitals. Rural populations have limited access to dermatologists and experienced HS diagnosticians, thus rendering the number of HS hospitalizations very low [36]. Urban teaching hospitals are more likely to have experts in HS, including primary care physicians and dermatologists, increasing the availability of treatment and care, thereby increasing the number of hospitalizations as well. Similarly, we found that large hospitals showed the highest percentage of HS hospitalizations (62.5%). Large hospitals can provide medical care to a greater number of patients, statistically increasing the chances of HS diagnosis and hospitalization. Additionally, large hospitals have greater access to diagnostic tools (such as MRI) and experts knowledgeable of HS diagnostic criteria, which may also increase the rate of diagnosis and hospitalization of HS [38]. Large hospitals and urban teaching hospitals may also have access to recent advances in treatment and larger hospitalization capacities, which would also result in an increased percentage of HS hospitalizations [39].

Our study indicated that hypertension, skin and subcutaneous tissue infections, and diabetes mellitus were the most common comorbidities associated with HS. A 2020 meta-analysis of observational studies indicated that cardiovascular risk factors, such as hypertension, present at a significantly higher rate in patients with HS [40]. Skin and subcutaneous tissue infections such as disorders of follicular occlusions (acne conglobata, dissecting cellulitis of the scalp, and pilonidal cyst) are common comorbidities of HS, as well as genetic keratin disorders associated with follicular occlusion [41]. In a study on the prevalence of type 2 diabetes mellitus (T2DM) among HS patients in the US, results indicated that HS was associated with T2DM across all demographic subgroups [42]. The study proposed that insulin resistance may predispose an individual to HS via follicular occlusion likely resulting from the mammalian target of rapamycin complex 1 (mTORC1) overactivity in the context of hyperinsulinemia [42]. Our study found morbid obesity to be another common co-existing condition, which is concurrent with previous studies in which obesity was found to be a comorbidity of HS [27,32,33,36,38-40]. In patients who present with both obesity and HS, lifestyle changes are incorporated into treatment plans alongside medical therapy [27,36]. We found cancer to also be a common comorbidity of HS. However, a relationship between HS and cancer has not been established and is debated, which warrants further study into their association [43]. Furthermore, the results of our study indicated that 12.7% of patients hospitalized with HS had a major or extreme loss of function and 3.5% were at a major or extreme likelihood of dying. A population-based cohort study of HS in the US concluded that HS appears to confer an independent risk of all-cause mortality [44]. As for the loss of function in patients with HS, there is insufficient literature regarding this association, substantiating the need for further research into this relationship.

Our study has several limitations. The study cohort was selected using ICD-9/ICD-10-CM diagnoses codes. There could be coding inaccuracies while using this huge database. Also, we only looked at the inpatient HS population given that the NIS does not collect data on outpatient visits. Despite these limitations, there are several strengths. This is the first nationwide study looking at the inpatient data on patient and hospital-level characteristics, the severity of illness, and rates of common comorbidities (like T2DM, obesity, hypertension, and skin infections) associated with HS.

## Conclusions

HS disproportionately affects young adults, women, and Black patients. The rate of HS hospitalizations per 100,000 all-cause hospitalizations showed an upward trend from 2008 to 2017. The overall male-to-female ratio during the study period was noted to be 1:1.30. Patients belonging to the lowest median household income quartile had the highest preponderance of developing HS. A significant proportion of these patients are at a major risk of major loss of bodily function or death. Prospective studies are needed to identify the risk factors for hospitalizations in these patient populations and devise appropriate prevention and treatment strategies.

## Appendices

Supplement 1: ICD codes

Hidradenitis Suppurativa

ICD-9 = 705.83; ICD-10 = L732.

Supplement 2: description of variables

Region

Regions are defined by the U.S. Census Bureau as below (Table 5).

**Table 5:** Regions as defined by the U.S. Census Bureau

Region	States
Northeast	ME, NH, VT, MA, RI, CT, NY, NJ, and PA
Midwest	OH, IN, IL, MI, WI, MN, IA, MO, ND, SD, NE, and KS
South	DE, MD, DC, VA, WV, NC, SC, GA, FL, KY, TN, AL, MS, AR, LA, OK, and TX
West	MT, ID, WY, CO, NM, AZ, UT, NV, WA, OR, CA, AK, and HI

Insurance Status

The expected primary payer for the hospitalization accounts for insurance status in our analysis (Table 6).

**Table 6:** Insurance status

Primary payer	Description
Medicare	Fee-for-service and managed care Medicare patients
Medicaid	Fee-for-service and managed care Medicaid patients
Private insurance	Blue Cross, Blue Shield, HMO (Health Maintenance Organization) plans, PPO (Preferred Provider Organization) plans, and other private insurance providers
Uninsured	Self-pay/no-charge

APR-DRG Severity of Illness

The All Patient Refined Diagnosis Related Group (APR-DRG) is a severity measurement system (Table 7). The groups are assigned using software developed by 3M Health Information Systems. Please see the link: https://www.hcup-us.ahrq.gov/db/vars/aprdrg/nisnote.jsp.

**Table 7:** All Patient Refined Diagnosis Related Group (APR-DRG) severity of illness

0	No class specified
1	Minor loss of function (includes cases with no comorbidity or complications)
2	Moderate loss of function
3	Major loss of function
4	Extreme loss of function

APR-DRG Risk of Mortality

The APR-DRG risk of mortality is shown below (Table 8).

**Table 8:** All Patient Refined Diagnosis Related Group (APR-DRG) risk of mortality

0	No class specified
1	Minor likelihood of dying
2	Moderate likelihood of dying
3	Major likelihood of dying
4	Extreme likelihood of dying
